# Foot Prints in the Rock

**Published:** 1881-04

**Authors:** 


### POPULAR SCIENCE DEPARTMENT.

STOCKBRIDGE, MADISON CO., N. Y., *February 20, 1881.*

*Harvey L. Byrd, M. D.*

DEAR SIR:—The Postmaster of our place handed me a line from you asking for facts in regard to "foot prints" in rock near Munnsville, with the request that I should furnish the same. Please find enclosed a rude cut of the prints. Though soiled and crumpled (the only one I have) it may give you a tolerably correct idea of the prints. I intended to have taken plaster casts of the tracks before the annual flow of the fall rains should cover the face of the rock, which is usually dry in the summer months. But for this bit of pastime however desirable, the farmer finds little leisure. An undefinable feeling comes over one as he stands by the foot prints, especially if he have little faith in the various theories of their origin. It seemed to me as if Nature, to torture us with intense curiosity, had turned down only a bit—a corner of the title page to her great volume upon which these mysterious impressions are found, while upon the lips of poor mortals who would fain probe the mysteries within she has set the seal of silence.

Some think these foot prints are formed by the action of water, but it is hard to resist the conviction that they are veritable human foot prints. The rock is given by the accompanying circular as unfossilifferous. If this term denotes an entire absence of vegetable and animal organism, the rock in question can hardly come under that term, for the most distinct impressions of shells have been found in gray limestone.

Yours very truly,

C. G. LYMAN.

### FOOT PRINTS IN THE ROCK.

There is, perhaps, nothing in this section of country that would prove as interesting to the scientific student as that presented in this sketch. About one-half mile from Munnsville, Madison Co., N. Y., at a place called, unpoetically, "Mosquito Point," is found the archælogical wonder to which we call your attention. The road from the station winds pleasantly around the foot of a hill, on the left you pass a perpendicular ledge of gray limestone, which in the summer is handsomely decorated with honey-suckle and other running vines, giving it the appearance of an ancient castle sadly out of repair. Large fragments of rock lie at its foot as though some giant in his might and wrath had hurled them there, from its battlements, at an approaching foe. A little stream crosses the road just beyond, which, when swollen by rains, goes tumbling over a fall of 15 feet perpendicular, but usually the stream is dry and the student has an excellent opportunity for investigation. On the brow of the precipice, in the bed of the creek, we find the foot prints, twelve in number, human and animal. The human foot was covered by a moccasin. Two persons had crossed the rock and one returned. This fact is ascertained from the difference in the size of the feet. The following is a description of the different prints: 1. The largest human foot print shows plainly the right foot, is 9 inches long, 3 inches wide and 1¼ deep. 2. On the right and above No. 1 is 6 inches long and about 3 inches wide, not quite so distinct. 3. Is the fellow of No. 2, both going in the same direction. 4. The same size as No. 1 going in the opposite direction. No. 5 was probably made by the smaller person and is going in still another direction. The size of the rock is 5x3½ feet which accounts for absence of pairs. The animal foot prints are very plain, their principal characteristics being a round toe, like that of a horse, and a notch in the heel, very unlike a horse. They are all the same size, about 3½ inches in diameter and 4 deep. There is another foot print, which is seen near the center of Fig. 1, which may be that of a very large animal, a saurian, perhaps, of the Post Tertiary. It is not very distinct and may have had its origin in another way. Fig. 2 shows the two foot prints enlarged. The rock is of gray limestone, unfossiliferous and very hard. The rocks are of the Upper Silurian and lie immediately upon the Marcellus Shale of the Hamilton Period. There can be but little doubt that prehistoric man has left his foot prints in mud that subsequently became rock. He may have stood here thousands of years ago and viewed the beautiful scenery which the valley affords.

NOTE.—We have not copied the cuts accompanying the above circular, because of their indistinctness, but will those our correspondent proposes to take as soon as he can make us acquainted with them.—EDS. INDEPENDENT PRACTITIONER.

